# Exploring the Longitudinal Links Popularity Goals and Adolescent Cyberbullying Perpetration: The Moderating Effects of Gender and Cultural Context

**DOI:** 10.3390/children11111302

**Published:** 2024-10-28

**Authors:** Michelle F. Wright

**Affiliations:** Department of Psychology, Indiana State University, Terre Haute, IN 47809, USA; michelle.wright@indstate.edu; Tel.: +1-812-237-2446

**Keywords:** cyberbullying, popularity, popularity goals, culture, gender

## Abstract

Background/Objectives: This longitudinal study aimed to examine how gender influences the relationship between popularity goals and cyberbullying perpetration among adolescents in China and the United States, controlling for peer-nominated popularity. Additionally, the study sought to identify potential cross-cultural differences in these dynamics. Methods: The research involved 1063 eighth and ninth graders (ages 12–16; 48.7% girls) who completed self-reported surveys in the fall of 2022 (Time 1) regarding cyberbullying behaviors and popularity goals. Peer nominations of popularity were also collected. Follow-up data on cyberbullying perpetration were obtained one year later in the fall of 2023 (Time 2). Results: Popularity goals were found to positively predict cyberbullying perpetration at Time 2 across all participants even after accounting for peer-nominated popularity. In China, this association was more pronounced among boys, whereas in the United States, the relationship was stronger for girls. Conclusions: Although cultural differences in the overall patterns were minimal, gender emerged as a significant moderating factor, revealing distinct cross-cultural variations. These findings underscore the need for culturally tailored approaches in interventions targeting adolescent cyberbullying.

## 1. Introduction

There has been limited research examining the influence of peer-related factors on cyberbullying, particularly the ways in which culture and gender may impact these relationships. This one-year longitudinal study aims to address these gaps by investigating how gender moderates the connection between peer-related factors, such as popularity goals, and cyberbullying behaviors among adolescents from both China and the United States. The study’s unique contribution lies in its longitudinal design and comparative analysis across cultural contexts.

### 1.1. Cyberbullying Perpetration

Cyberbullying refers to the intentional use of information and communication technologies—such as smartphones, social media, and online gaming platforms—to harm, intimidate, or threaten others [1]. Common forms of cyberbullying include sending or receiving hurtful messages, spreading rumors, name calling, and participating in gossip, either as a target or instigator. Concerns about adolescent involvement in cyberbullying stem from numerous studies linking it to negative outcomes such as depression, anxiety, loneliness, academic struggles, and increased rates of school absenteeism or truancy [2]. Additionally, individuals who engage in cyberbullying often display traits such as low empathy, a greater tolerance for cyberbullying behaviors, and psychopathic tendencies [3]. While much of the research on adolescent cyberbullying has focused on regions like Europe, Australia, and North America [2,4], interest in this issue has been growing in Asian countries as well [5]. With China leading the world in internet users, followed by India and the United States, further studies are crucial to deepen the understanding of cyberbullying in non-Western cultures. This is particularly important as increased internet access exposes adolescents to heightened risks of both perpetrating and being victimized by cyberbullying [6].

### 1.2. Culture

In collectivistic cultures like China, the self-concept is interdependent, fostering attitudes and behaviors that prioritize group harmony and social connections. This cultural framework also includes a strong emphasis on relational hierarchies, which may heighten adolescents’ susceptibility to cyberbullying [7]. In contrast, individualistic cultures, such as that of the United States, promote an independent self-concept, where personal goals and self-expression take precedence. Generally, collectivist values are associated with lower levels of cyberbullying, while individualistic values correlate with higher rates of involvement [8,9]. Gender roles, shaped differently across cultures, also influence cyberbullying participation. Studies from Western countries have yielded mixed results on gender differences in both cyberbullying victimization and perpetration [10,11]. However, research in China consistently shows that boys report higher involvement in cyberbullying than girls [12,13,14]. Few studies have explored the combined effects of gender and cultural differences on cyberbullying. One such study revealed that boys in both India and China are more likely than girls to engage in cyberbullying perpetration [15]. For a comprehensive understanding of cyberbullying, researchers must consider the influence of both culture and gender. Additionally, future research should investigate how cyberbullying involvement varies between individual and peer-related contexts.

### 1.3. Popularity Goals

Adolescence is a critical time for developing goal-oriented behaviors, particularly those related to gaining social status [11]. During this period, adolescents often set goals to boost their popularity (referred to as popularity goals) or to increase their social acceptance among peers (known as social preference goals) [16]. Research suggests that popularity goals are frequently linked to aggressive behaviors, whereas social preference goals tend to be negatively associated with aggression [11]. Cultural values may influence how popularity goals are prioritized and connected to cyberbullying across different countries. In collectivistic cultures, where relational hierarchies are emphasized, adolescents may pursue popularity goals to enhance their social standing [7]. Conversely, in individualistic cultures, where personal interests are often prioritized over group interests, adolescents may also be driven by popularity goals. The relationship between gender, popularity goals, and cyberbullying remains unclear. Some studies suggest that girls, being more relationally focused than boys, might be more inclined to seek peer status through popularity goals [17]. However, these effects are likely to vary by country, as cultural norms influence gender roles differently. For example, girls in collectivistic cultures who pursue popularity goals may be at lower risk of cyberbullying involvement compared to those in individualistic cultures, while the opposite pattern could be seen among boys. Moreover, it is important to examine these associations while considering peer-nominated popularity, as peer-recognized popularity is also linked to cyberbullying perpetration [18]. Future research should explore how status-related goals relate to cyberbullying behavior while controlling for adolescents’ existing levels of popularity.

### 1.4. Present Study

The primary goal of this study was to examine how gender moderates the relationships between Machiavellianism, popularity goals, and cyberbullying perpetration among adolescents in China and the United States. Given that girls often prioritize relational goals more than boys, their pursuit of popularity may differ by gender, and these differences may further vary across cultural contexts [17]. Previous research has yielded mixed findings on gender differences in cyberbullying perpetration in individualistic cultures [10,11], while more consistent patterns have been observed in collectivistic cultures [12,13,14]. The distinct contribution of this study lies in its longitudinal, cross-cultural design and its focus on gender disparities. To guide the research, the following questions were posed:

1. What is the relationship between popularity goals and cyberbullying perpetration over a one-year period?
2. How do gender and culture influence the associations between popularity goals and cyberbullying perpetration during that same time frame?

## 2. Methods

This study involved 1063 eighth and ninth-grade adolescents from China (n = 533; 48.3% girls) and the United States (n = 530; 50.3% girls), with participants ranging in age from 12 to 16. Overall, 49.1% of the sample identified as female. The Chinese participants were recruited from three middle schools in Beijing, while the U.S. participants came from six middle schools in the Midwest. No data on income and family background were collected for this study.

### 2.1. Procedures

Ethical approval for the study was granted by DePaul University (PSY20120606, 6 June 2012). In both countries, school principals provided consent for data collection. Meetings were held with principals, teachers, and researchers to discuss the study’s objectives and the procedures for adolescent participation. Classroom announcements were made, and parental consent forms were distributed to students, who returned them to their schools after obtaining the required signatures. Data collection began in the fall of 2022 (Time 1), when the adolescents were in the 7th or 8th grade. On the day of data collection, students gave their own assent to participate, and none declined. They completed questionnaires that measured their peer-nominated popularity, cyberbullying perpetration, and popularity goals. All study materials were translated into Mandarin for the Chinese participants and backtranslated by research assistants to ensure accuracy.

In the fall of 2023 (Time 2), a year later, a reminder letter was sent home with the students. Parents or guardians who did not wish their child to participate were asked to write the child’s name on the letter, which the student would then return to school. No letters were returned. Approximately 25 students were unavailable for Time 2 data collection due to reasons such as relocation or illness. During this second round of data collection, the adolescents—now in the 8th or 9th grade—completed a follow-up questionnaire focused on cyberbullying perpetration.

### 2.2. Measures

#### 2.2.1. Gender

Adolescents were asked to select their gender from the options “male”, “female”, or “other”. None of the participants selected the “other” option.

#### 2.2.2. Cyberbullying Perpetration

Cyberbullying perpetration was measured by asking adolescents how often they engaged in specific cyberbullying behaviors during the current school year [11]. The questionnaire consisted of nine items related to perpetration (e.g., “How often do you call a peer mean names online or through text messages?”). Responses were rated on a scale from 1 (never) to 5 (all of the time). Final scores for both cyberbullying victimization and perpetration were calculated by averaging the respective items. McDonald’s omega demonstrated strong reliability at both Time 1 (China: ω = 0.92; United States: ω = 0.92) and Time 2 (China: ω = 0.90; United States: ω = 0.90).

#### 2.2.3. Popularity Goals

Popularity goals were measured using the Popularity Goals Subscale from the Social Status Goals questionnaire [19]. This subscale included six items, such as “I want to be popular among my peers”. Participants rated their responses on a scale from 1 (never) to 5 (all the time). Popularity goals were assessed at Time 1 only with McDonald’s omega indicating reliability scores of 0.87 for both China and the United States.

#### 2.2.4. Peer-Nominated Popularity

Adolescents’ popularity was assessed through peer nominations, following established methods from prior research [20]. Participants were asked two questions regarding popularity: who is popular and who is unpopular. The total number of nominations each participant received for both items was summed and then standardized within their grade using z-scores. To determine overall popularity, the standardized score for “unpopular” was subtracted from the standardized score for “popular” for each adolescent with the resulting difference restandardized within their grade.

### 2.3. Analytic Plan

To evaluate measurement invariance, a multigroup confirmatory factor analysis was conducted in Mplus Version 8, which confirmed that the measures were consistent and equivalent across the two countries. While detailed descriptions of the models are not included here due to space constraints, interested readers can request this information by contacting the author.

To address the research questions, a multigroup structural equation model was utilized, treating the two countries as separate groups. The Robust Maximum Likelihood estimator and the Full Information Maximum Likelihood method were employed to manage missing data, which constituted approximately 0.3% of the dataset (nine incomplete cases). The Little’s Missing Completely at Random (MCAR) test revealed no systematic bias in the missing data, χ^2^ = 52.69, df = 91, *p* = n.s.

The model included paths from popularity goals to Time 2 cyberbullying perpetration as well as paths from gender to both popularity goals and Time 2 cyberbullying perpetration. Interaction terms between popularity goals and gender were also examined. Additionally, Time 1 cyberbullying perpetration was allowed to predict Time 2 cyberbullying perpetration, and peer-nominated popularity was permitted to predict popularity goals to control for achieved status. Simple slope analyses were performed to further investigate these interactions.

## 3. Results

Correlations were computed among all variables across the different countries (see Table 1), and the findings aligned with expectations. In particular, both popularity goals and peer-nominated popularity were positively correlated with cyberbullying perpetration at both Time 1 and Time 2.

The multigroup analysis demonstrated a satisfactory fit for the model, χ^2^ = 615.33, df = 600, CFI = 0.99, TLI = 0.99, RMSEA = 0.04, SRMR = 0.04 (see Table 2). Popularity goals were positively associated with Time 2 perpetration while controlling for peer-nominated popularity. Additionally, peer-nominated popularity showed a positive relationship with popularity goals. Among Chinese adolescents, gender emerged as a significant predictor of Time 2 cyberbullying perpetration with boys being more likely to engage in such behaviors compared to girls. However, no significant gender differences in cyberbullying perpetration were observed among U.S. adolescents.

Significant two-way interactions were identified between popularity goals and gender in relation to Time 2 cyberbullying perpetration among adolescents in China while controlling for peer-nominated popularity. The association between popularity goals and Time 2 cyberbullying perpetration was significantly stronger for boys (China: β = 0.29, *p* = 0.001) compared to girls (China: β = 0.05, *p* = n.s.). Similarly, among adolescents in the United States, a two-way interaction between popularity goals and gender was observed with respect to Time 2 cyberbullying perpetration. In the U.S., the relationship was stronger for girls (β = 0.27, *p* = 0.005) than for boys (β = 0.04, *p* = n.s.).

## 4. Discussion

This one-year longitudinal study examined the moderating role of gender in the relationships between popularity goals and Time 2 cyberbullying perpetration among adolescents from China and the United States. The findings highlight the importance of considering peer-related risk factors, such as popularity goals, which may increase the likelihood of cyberbullying among adolescents. Results showed that popularity goals were positively linked to cyberbullying perpetration in both Chinese and U.S. adolescents, suggesting that these risk factors may have universal implications for cyberbullying behavior.

Popularity goals were directly associated with Time 2 cyberbullying perpetration. Previous studies have established connections between popularity goals and aggressive behaviors in offline contexts [19] as well as with both popularity and cyberbullying perpetration [18]. While the link between popularity goals and cyberbullying has been less explored, evidence suggests that offline aggression and cyberbullying share similar characteristics [11]. The current findings extend this research by indicating that adolescents with strong popularity goals, regardless of cultural background, may engage in cyberbullying to enhance their social standing within peer groups, particularly in their pursuit of higher status.

Among Chinese adolescents, gender was a significant predictor of Time 2 cyberbullying perpetration with boys more likely to engage in cyberbullying than girls even after accounting for prior involvement. This pattern was not found among U.S. adolescents. Previous research suggests that boys in collectivist cultures are more involved in cyberbullying [12,13,14], whereas gender differences in cyberbullying perpetration within individualistic cultures, like the U.S., tend to be less consistent. The present study found no significant gender differences in cyberbullying perpetration among U.S. adolescents, which is consistent with prior findings [10,11].

Although the primary effects of popularity goals on cyberbullying perpetration were similar across both countries, gender moderated these relationships differently in China and the U.S. In China, boys showed stronger positive associations between popularity goals and cyberbullying perpetration, aligning with research that suggests boys in collectivist cultures are more likely to engage in competitive behaviors to achieve higher social status [7]. In contrast, for U.S. adolescents, gender moderated the relationship between popularity goals and cyberbullying perpetration with a stronger association observed for girls. In individualistic cultures, where peer status is highly valued, girls may face added pressure from media and peers to elevate their social standing, contributing to their involvement in cyberbullying.

### Limitations and Future Research Directions

The sample exhibits low variability, as it primarily consists of students from a limited number of schools—in this case, three schools in Beijing and six schools in the United States. This context-specific nature may limit the generalizability of the findings to broader populations. Another limitation of this study is the absence of an evaluation of adolescents’ cultural values, which could elucidate intracultural differences in the connections between popularity goals and cyberbullying perpetration. Future research should include assessments of both adolescents’ and their parents’ cultural values to provide a more comprehensive understanding of how these values might moderate the examined associations. Furthermore, additional studies should investigate the various types of cyberbullying behaviors and their expressions across different technologies. Exploring the motivations driving cyberbullying, such as revenge or the pursuit of status, could also enrich our understanding of these relationships. Additionally, the use of Likert-type scales for measuring cyberbullying and popularity presents its own challenges. For instance, the measure of cyberbullying may not fully capture the complexities of such behavior. These factors can significantly influence the results and the interpretation of the data, warranting caution in drawing conclusions.

## 5. Conclusions

Although few cultural differences were observed in the main effects, the moderating influence of gender revealed important cross-cultural variations. These findings emphasize the necessity of understanding cyberbullying within diverse contexts, which can aid in creating culturally sensitive interventions aimed at reducing cyberbullying involvement among adolescents.

## Figures and Tables

**Table 1:** Correlations among popularity, popularity goals, and Time 1 and Time 2 cyberbullying perpetration.

	1	2	3	4
1. Peer-Nominated Popularity	---	---	---	---
2. Popularity Goals	0.32 ***/ 0.41 ***	---	---	---
3. T1 CBP	0.31 ***/ 0.35 ***	0.33 ***/ 0.38 ***	---	---
4. T2 CBP	0.31 ***/ 0.37 ***	0.30 ***/ 0.37 ***	0.42 ***/ 0.41 ***	---
*M* (*SD*)	3.50 (1.00)/ 3.67 (0.91)	3.00 (0.80)/ 3.25 (0.88)	2.66 (0.85)/ 2.69 (0.93)	2.69 (0.86)/ 2.73 (0.88)

Note. T1 = Time 1; T2 = Time 2; CBP = cyberbullying perpetration; the top number is the correlation for Chinese adolescents, and the second number is for U.S. adolescents. *** *p* < 0.001.

**Table 2:** Structural regression model results.

Country	Predictors	Time 2 Cyberbullying Perpetration	
		β	SE
China	Popularity Goals (PG)	0.28 **	0.08
	Gender	−0.22 *	0.03
	PG x Gender	−0.14 **	0.05
United States	Popularity Goals	0.31 ***	0.11
	Gender	0.08	0.02
	PG x Gender	0.14 **	0.05

* *p* < 0.05. ** *p* < 0.01. *** *p* < 0.001.

## Data Availability

The data presented in this study are available on request from the corresponding author due to privacy reasons.
